# SnS_2_ Nanocrystalline-Anchored Three-Dimensional Graphene for Sodium Batteries with Improved Rate Performance

**DOI:** 10.3390/nano10122336

**Published:** 2020-11-25

**Authors:** Li Zeng, Liping Zhang, Xingang Liu, Chuhong Zhang

**Affiliations:** 1 Polymer Research Institute, Sichuan University, Chengdu 610065, China; zengli_0718@foxmail.com (L.Z.); li_ping_zhang@foxmail.com (L.Z.); 2 State Key Laboratory of Polymer Materials Engineering, Sichuan University, Chengdu 610065, China

**Keywords:** sodium ion batteries, SnS_2_ nanocrystalline, three-dimensional porous graphene

## Abstract

Tin disulfide (SnS_2_) is regarded as one of the most suitable candidates as the electrode material for sodium-ion batteries (SIBs). However, the easy restacking and volume expansion properties of SnS_2_ during the charge/discharge process lead to the destruction of the electrode structure and a decrease in capacity. We successfully synthesized a SnS_2_ nanocrystalline-anchored three-dimensional porous graphene composite (SnS_2_/3DG) by combining hydrothermal and high-temperature reduction methods. The SnS_2_ nanocrystalline was uniformly dispersed within the connected reduced graphene oxide matrix. The SnS_2_/3DG battery showed a high reversible capacity of 430 mAh/g after 50 cycles at 100 mA/g. The SnS_2_/3DG composite showed an excellent rate capability with the current density increasing from 100 mA/g to 2 A/g. The excellent performance of the novel SnS_2_/3DG composite is attributed to the porous structure, which not only promoted the infiltration of electrolytes and hindered volume expansion for the porous structure, but also improved the conductivity of the whole electrode, demonstrating that the SnS_2_/3DG composite is a prospective anode for the next generation of sodium-ion batteries.

## 1. Introduction

The demand for high-performance electrochemical energy storage and energy conversion devices is ever-growing [1,2,3]. Although lithium ion batteries (LIBs) have been widely adopted, lithium sources are limited, which makes meeting the ever-increasing demand for large-scale applications difficult [4]. Similar to the electrochemical reaction mechanism of LIBs, sodium-ion batteries (SIBs) are considered one of the most promising alternatives to LIBs due to the abundant sodium resources and their low cost [5,6]. Significant effort has been invested in developing advanced electrode materials to improve the performance and practical value of SIBs [7,8]. SnS_2_ has been widely used as an anode material for SIBs due to its high theoretical specific capacity, large interlayer spacing, unique layered structure, and environmental friendliness [9]. The octahedral coordination structure is formed by each Sn atom connected with eight S atoms through covalent bonds. The layers of SnS_2_ interact with each other through van der Waals forces [10,11]. The interlayer spacing also provides a pathway for the migration of ions. However, like other transition metals (e.g., oxides and sulfides), SnS_2_ suffers from low conductivity. The volume expansion effect (~420%) can cause damage as it leads to the serious pulverization of the crystal structure [12,13]. Researchers have reported that the volume expansion effect of SnS_2_ materials could be mitigated by nanosizing to improve the electrochemical properties of SnS_2_ materials, such as SnS_2_ nanorods [14,15], SnS_2_ nanosheets [16,17], SnS_2_ nanoflowers [18,19], etc. However, nanomaterials tend to agglomerate, and the high specific surface area of nanomaterials may cause side reactions with the electrolyte and thick solid electrolyte interphase (SEI) films may form on the surface of the materials, which seriously affects the cycle performance and rate performance of the electrode. 

Compositing with carbon materials is also an effective method to improve the electrochemical performance of SnS_2_ [7]. Among these, graphene stands out due to its excellent mechanical, thermal, and electron transfer ability properties [20]. The introduction of graphene is supposed to effectively prevent the stacking of SnS_2_ nanosheets as the van der Waals force between adjacent layers of SnS_2_ is destroyed by graphene, which is favorable for the electron transfer rate [21,22,23]. To further improve the electrochemical performance of SnS_2_ as the SIB anode, various structures of SnS_2_/graphene hybrids have been designed [23,24]. In addition, controlling the morphology and particle size of the material can improve its electrochemical performance. Among the methods of synthesizing SnS_2_/graphene, the in-situ hydrothermal process is the most common. This method can simultaneously control the structure of composite materials and the morphology and particle size of SnS_2_ [25]. However, due to the limitation of synthesis conditions (low pressure and temperature), the degree of carbonization of graphene is low, which seriously affects the rate performance of composite materials. For instance, Liu et al. successfully prepared few-layer SnS_2_/graphene composites with a high capacity of 521 mAh/g (at 0.05 A/g) by stripping the commercial SnS_2_ particles with graphene oxide via the hydrothermal reaction. However, the capacity was only 165 mAh/g at 2 A/g [26].

In this study, to simultaneously achieve a high capacity and a high rate, we used an effective method that combines the hydrothermal and high-temperature processes to prepare a SnS_2_ nanocrystalline anchored 3D porous graphene composite. The presence of graphene can effectively inhibit the volume expansion effect of SnS_2_ and improve the conductivity of the SnS_2_/3DG composite. The two-step reduction method significantly improves the reduction degree of graphene, resulting in an enhancement in the rate performance of the SnS_2_/3DG composite.

## 2. Materials and Methods

### 2.1. Preparation of the SnS_2_ Nanocrystalline

We added 10 mmol of tin(IV) chloride pentahydrate (SnCl_4_·5H_2_O) (Aldrich, ≥99.9%, St. Louis, MO, USA) and 10 mmol of anhydrous citric acid (C_6_H_8_O_7_) (Aldrich, ≥99.9%) to 80 mL of deionized water and stirred for 1 h. Another 10 mmol of thioacetamide (CH_3_CSNH_2_) (Aldrich, ≥99.5%, St. Louis, MO, USA) was added to the mixture after SnCl_4_·5H_2_O and C_6_H_8_O_7_ were completely dissolved. The mixture was stirred for an additional 30 min. Next, the well-dissolved mixture was transferred into a 100 mL hydrothermal reaction kettle and reacted at 130 °C for 12 h. After the reactor cooled to room temperature, the obtained SnS_2_ precipitate was centrifuged with deionized water and ethanol.

### 2.2. Preparation of SnS_2_/3DG Composite

The prepared SnS_2_ dispersion with a concentration of 80 mg/mL was treated with ultrasonic dispersion for 6 h. The graphite oxide powder (GO, Changzhou No.6 element Co., Ltd., Chang Zhou, China) was dispersed in deionized water (the preparation concentration was 4 mg/mL) and the graphene oxide suspension was obtained after ultrasonic stripping for 1 h. Next, 20 mL of the GO suspension was added into the reaction bottle and 1 mL of SnS_2_ dispersion was added with a pipette gun under ultrasonic conditions. After continuous ultrasonic dispersing for 30 min, 320 mg of vitamin C (VC) (Aldrich, 99.7%, St. Louis, MO, USA) was added to the reaction bottle. The mixed solution in the reaction bottle was placed in the 80 °C water bath after the VC was dissolved evenly. The mixture hydrothermally reacted for 8 h to obtain a hydrogel with a monolith structure. The hydrogel was then freeze-dried to obtain the three-dimensional porous graphene-loaded SnS_2_ composite (SnS_2_/3DG). Finally, the SnS_2_/3DG was placed in a tubular furnace and thermally treated at 400 °C for 2 h in an argon atmosphere. 

### 2.3. Characterizations

The microstructure of the material was characterized by a Philips FEI Quanta (Hillsboro, OR, USA) 200F high-resolution scanning electron microscope (SEM) and transmission electron microscope (TEM). The phase analysis was carried out by a Rigaku smart-lab III X-ray diffraction (XRD), using a Cu-Kα radiation source. The proportion of each component in the composite was analyzed by a thermogravimetric (TG) analyzer (TG209F1, Netzsch, Selbu, Germany). The Raman spectra of the materials were analyzed by a Horiba LabRAM HR spectrometer (Pasadena, CA, USA).

For the electrochemical characterization, the SnS_2_/3DG composite powder, conductive carbon black, and adhesive polyvinylidene fluoride (PVDF) were mixed with a mass ratio of 8:1:1, and a certain amount of N-methylpyrrolidone (NMP) was used as solvent to grind and prepare uniform slurry. The slurry was coated on the pretreated clean copper foil collector and dried in a vacuum oven at 100 °C for 12 h. The CR2032 button cell was assembled in the glove box, as the sodium plate was used as the counter electrode and a glass fiber (GF/D, Whatman, Buckinghamshire, UK) porous membrane was used as the separator. A 1 M ethylene carbonate (EC) and dimethyl monocarbonate (DMC) solvent with a volume ratio of 1:1, which was dissolved with sodium perchlorate (NaClO_4_), was used as the electrolyte. The cyclic voltammetry (CV) of the cell was characterized by a VMP3 (Biologic, Paris, France). The electrochemical impedance spectroscopy (EIS) was measured on a VMP3 workstation with an amplitude of 5 mV and a frequency range of 0.01–100,000 Hz.

## 3. Results and Discussion

### 3.1. Structure and Morphology Analysis of SnS_2_ and SnS_2_/3DG Composites

**Figure 1:**
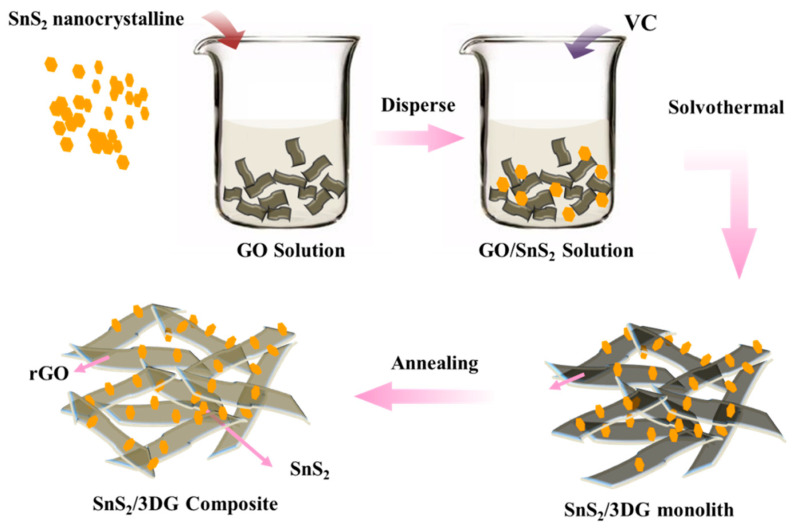
Schematic illustration of the preparation of the SnS_2_/3DG composite.

The SnS_2_/3DG composite was fabricated on the basis of the hydrothermal method followed by a controllable, low-temperature water bath process. As illustrated in Figure 1, the pure SnS_2_ particles were obtained by mixing CH_3_CSNH_2_ and SnCl_4_·5H_2_O. The adsorbed Sn^4+^ ions reacted with the gradually released H_2_S from the decomposition of CH_3_CSNH_2_ during the hydrothermal process [27], while SnS_2_ uniformly nucleated to form nanoparticles. Next, the prepared SnS_2_ nanoparticles were immersed into the well-dispersed GO suspension, and a mild solvothermal method with the adding of reducing agent was introduced to not only efficiently disperse the SnS_2_ nanoparticles but also to reduce GO. Finally, the porous SnS_2_/3DG composite was successfully fabricated by the freeze-drying and thermal reduction step. This unique 3D porous structure facilitates the migration of Na^+^ and electrons and benefits the high sodium storage performances.

**Figure 2:**
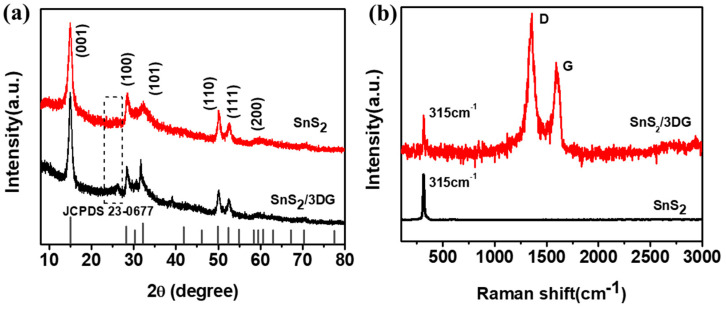
(**a**) XRD patterns of the pristine SnS_2_ and the SnS_2_/3DG composite; (**b**) Raman analysis of the pristine SnS_2_ and the SnS_2_/3DG composite.

SnS_2_ nanocrystalline was successfully synthesized by the hydrothermal process and SnS_2_/3D composite was obtained by the low-temperature water bath method. Figure 2a shows the XRD patterns of the pure SnS_2_ nanocrystalline and the SnS_2_/3D composite. All diffraction peaks of pure SnS_2_ nanocrystalline corresponds well with the standard spectrum of hexagonal SnS_2_ (JCPDS 23-0677) [27] without any evident impurity. In addition, there is an extra diffraction peak near 2θ = 25° of the SnS_2_/3DG composite, corresponding to the typical (002) plane of graphene [28,29]. The Raman spectra of the pure SnS_2_ nanocrystalline and the SnS_2_/3DG composite are shown in Figure 2b. The characteristic peak of the SnS_2_ nanocrystalline near 315 cm^−1^ reveals the vibration modes of A1g of SnS_2_ [30]. In addition, there are two other well-defined peaks at 1355 and 1590 cm^−1^, which belong to the D and G peaks of graphene, respectively. The prominent D peak refers to the vibration caused by the defects of graphene, while the G peak refers to the in-plane vibration of the sp^2^ hybridized carbon atoms of graphene [31,32]. The intensity ratio of the D band to the G band (I_D_/I_G_) can show the disorder degree of the carbon material. The calculated I_D_/I_G_ value of the SnS_2_/3DG composite decreased to 1.29, which proves the high reduction degree of graphene after the thermal reduction process [33].

**Figure 3:**
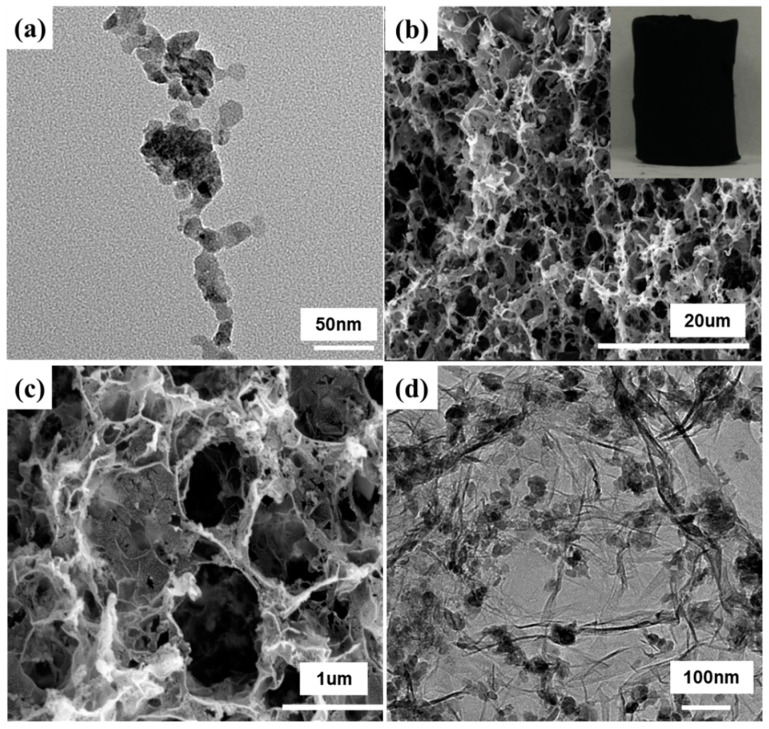
(**a**) TEM image of pristine SnS_2_ nanoparticles; (**b**,**c**) SEM images of the SnS_2_/3DG composite, inset: the photo of the SnS_2_/3DG monolith; (**d**) TEM image of the SnS_2_/3DG composite.

The morphologies of the SnS_2_ nanocrystalline and the SnS_2_/3DG composite are shown in Figure 3. The TEM image in Figure 3a reveals that the SnS_2_ nanocrystalline with an average diameter of 20 nm was seriously agglomerated, which is not conducive to maintaining good electrochemical stability. Figure 3b,c shows the SEM spectra of the SnS_2_/3DG composite at different magnifications where the SnS_2_/3DG composite displays obvious 3D porous morphology with the graphene sheets connecting to form a continuous conductive network. The complete monolith structure of the SnS_2_/3DG is shown in the inset of Figure 3b. Figure 3d shows the TEM spectrum of the SnS_2_/3DG composite, where SnS_2_ nanocrystalline particles are uniformly dispersed on the graphene sheets with no obvious agglomeration because the van der Waals force between the adjacent SnS_2_ is destroyed by well-dispersed graphene sheets [18]. The unique three-dimensional porous structure and the interaction between SnS_2_ nanocrystals and graphene provide a good possibility for the electrochemical performance of the SnS_2_/3DG composites.

**Figure 4:**
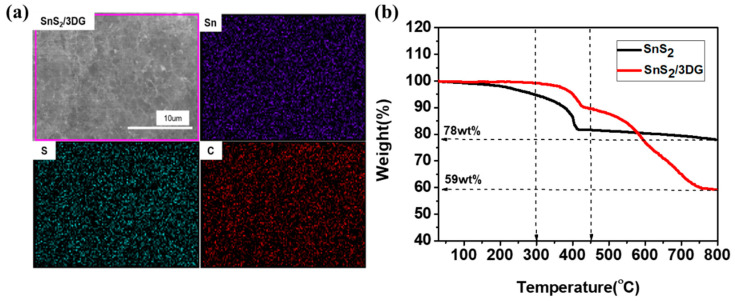
(**a**) EDS dot-mapping images of Sn, S, and C elements of the SnS_2_/3DG composite; (**b**) TG curves of the pristine SnS_2_ and the SnS_2_/3DG composite.

In addition, the EDS mapping in Figure 4a displays the good distribution of Sn, S, and C elements in the SnS_2_/3DG composite, which may guarantee the cycling stability. Figure 4b shows the TG curves of the pure SnS_2_ and the SnS_2_/3DG composite. For the pristine SnS_2_ sample, the mass loss in the range from room temperature to 300 °C occurred due to the evaporation of water molecules, while the mass loss over 300 to 800 °C represents the transformation from SnS_2_ into SnO_2_ [34]. The SnS_2_/3DG composite also displayed a mass loss in the temperature range of 25 to 300 °C, which was due not only to the evaporation of water molecules, but also to the decomposition of residual oxygen-containing functional groups in graphene. SnS_2_ gradually transferred to SnO_2_ from 300 to 450 °C, and the excess SnS_2_ was further oxidized and the graphene decomposed completely at the temperature range of 450 to 800 °C. The calculated content of SnS_2_ was 76 wt%.

### 3.2. Electrochemical Analysis of SnS_2_/3DG Composites

**Figure 5:**
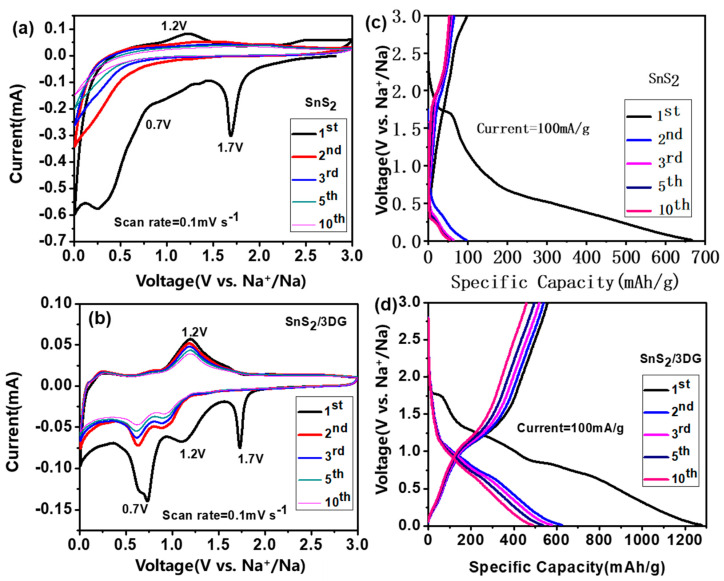
Cyclic voltammograms curves of (**a**) the pristine SnS_2_ and (**b**) the SnS_2_/3DG composite. The first, second, third, fifth, and tenth charge–discharge profiles of (**c**) the pristine SnS_2_ and (**d**) the SnS_2_/3DG composite.

To understand the sodium storage process of a SnS_2_/3DG anode, Figure 5a,b shows the cyclic voltammograms curves (CVs) of the pure SnS_2_ and the SnS_2_/3DG composite in the voltage range of 0.01–3.0 V. For the pure SnS_2_ material, the reduction peak near 1.7 V corresponds to the insertion of sodium ions into SnS_2_ nanocrystalline in the first discharge process of the pure SnS_2_ electrode (SnS_2_ + 4Na^+^ + 4e^−^ → Sn + 2Na_2_S_2_) [35], while the peak near 0.7 V is attributed to the synergetic conversion, the alloying reactions, and the formation of SEI films. The oxidation peak appears at 1.2 V for the first cycle of the charging process. However, there were no obvious oxidation and reduction peaks in the CV cycle of pure SnS_2_ since the second cycle, indicating the low capacity of pure SnS_2_. The reduction peak near 1.7 V was also found in the SnS_2_/3DG composite. Sn produced in the reduction provided the tin source for the Sn/Na alloying reaction at 1.2 and 0.7 V (Sn + xNa^+^ + xe^−^ → Na_x_Sn) [35], and the oxidation peak at 1.2 V similarly corresponds to the dealloying effect of the SnS_2_/3DG composite. Figure 5c displays the galvanostatic charge–discharge curves of the pure SnS_2_ and the SnS_2_/3DG composite at the current density of 100 mA/g. The SnS_2_/3DG composite delivered a specific initial discharge capacity and a charge specific capacity of 1276 and 557 mAh/g for the first cycle, respectively, which are much higher than those of the pure SnS_2_ nanocrystal electrode.

**Figure 6:**
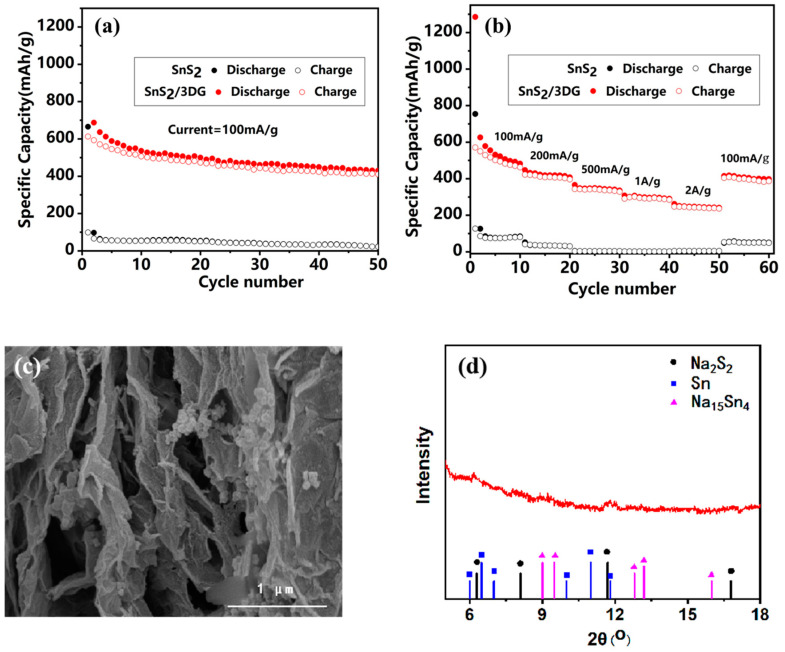
The cycling performance (**a**) and rate performance (**b**) of the pristine SnS_2_ and the SnS_2_/3DG composite; the morphology (**c**) and XRD (**d**) characterization after cycling.

The cycling performance of the pure SnS_2_ and the SnS_2_/3DG composite at the current density of 100 mA/g are shown in Figure 6a. After the composite with 3D graphene, the reversible specific capacity of the SnS_2_/3DG composite after 50 cycles improved to 430 mAh/g, while that of pure SnS_2_ was only 22 mAh/g. Figure 6b shows the rate performance of pure SnS_2_ and SnS_2_/3DG composites at various current densities. The SnS_2_/3DG composite delivered a reversible discharge specific capacity of 462 mAh/g at the current density of 100 mA/g, and the specific capacity decreased to around 407, 345, and 292 mAh/g as the current density gradually reached 200 mA/g, 500 mA/g, and 1 A/g, respectively. Even when the current density reached 2 A/g, the SnS_2_/3DG composite still displayed a steady discharge capacity of around 247 mAh/g, indicating an excellent rate. In addition, the reversible specific capacity of the SnS_2_/3DG composite returned to about 414 mAh/g when the current density was reset to 100 mA/g. However, the specific capacity of pure SnS_2_ was as low as 90 mAh/g at the current density of 100 mA/g, and the specific capacity of pure SnS_2_ almost dropped to 0 mA/g when the current density increased to 2 A/g. The specific capacity was only about 50 mAh/g when the current density returned to 100 mA/g.

It is the unique three-dimensional porous structure and the interaction between the SnS_2_ nanocrystalline and graphene that result in the composite having a strong electrochemical rate performance and a high capacity. The reasons why SnS_2_/3DG has high specific capacity and excellent rate performance are as follows: (1) The high-degree reduction of 3D graphene creates the continuous conductive matrix, which is beneficial for the rate performance; (2) the porous structure of SnS_2_/3DG is conducive to the diffusion of electrolyte and the diffusion rate of Na^+^ increases; (3) the volume expansion of the SnS_2_ nanocrystalline is restrained by the flexible graphene sheets, and the cycling stability is improved for the solid structure of the SnS_2_/3DG composite. A comparison of the performance of the SnS_2_/3DG composite with the literature is provided in Table 1. We also researched the post-mortem analysis after three cycles at 0.1 A/g. The morphology of the electrode after three cycles is shown in Figure 6c. The porous structure was preserved and did not undergo pulverization, and the thickness of the graphene layer increased for the formation of SEI films. The XRD result in Figure 6d shows that there are some new peaks belonging to Na_2_S_2_, Na_14_Sn_5,_ and Sn, which is consistent with the above charge–discharge process.

**Figure 7:**
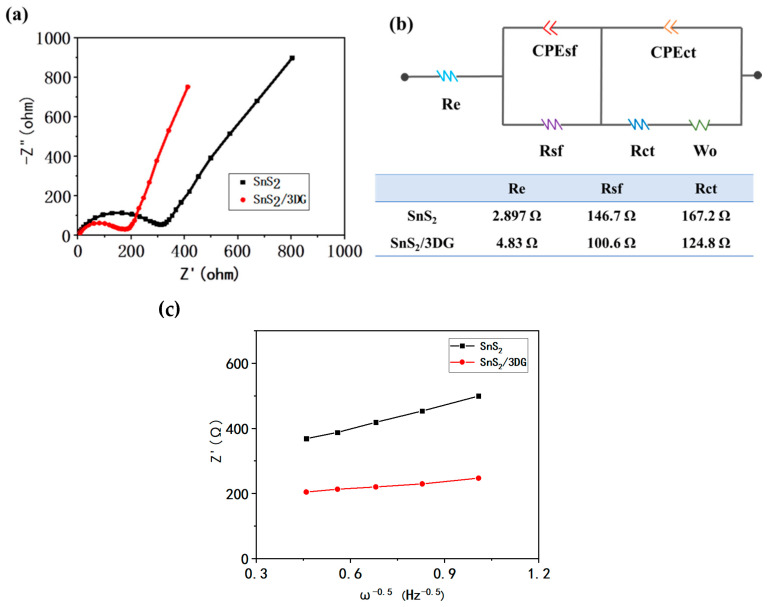
(**a**) Nyquist plots of the pristine SnS_2_ and the SnS_2_/3DG composite, (**b**) the equivalent circuit, and (**c**) the plot of σ_w_ as a function of ω^−1/2^.

The Nyquist spectra of the pure SnS_2_ and the SnS_2_/3DG composite are shown in Figure 7a. Both the pristine SnS_2_ and the SnS_2_/3DG composite showed semicircles in the high frequency region and a diagonal line in the low frequency region, which are related to the Warburg impedance. The equivalent circuit is shown in Figure 7b, where the symbol *Re* represents the electrolyte impedance contributed by current collectors, electrodes, separators, and the interface between electrodes and electrolytes. *Rsf* and *CPEsf* represent the resistance and capacity of the combination of migration and the interface impedance of SEI films, respectively. *Rct* and *CPEct* represent the charge transfer impedance and charge transfer capacitance, respectively. *Z_W_* is the Warburg impedance, which is usually revealed by the straight line in the low frequency region. The SnS_2_/3DG composite delivered a much lower total resistance of 230.2 Ω than the pristine SnS_2_ (316.8 Ω), which is also lower than the hydrothermal-treated flower-like SnS_2_/graphene battery [26], confirming that the conductivity was improved. The fast electron transport facilitates the redox reaction. The ion diffusion property can also affect the electrochemical performance of the battery. The Warburg coefficient (σ_w_) was calculated by the EIS results, as the σ_w_ is the slope of the function of real resistance (Z’) and ω^−1/2^ (Figure 7c). The SnS_2_/3DG electrode delivered a much smaller Warburg coefficient than the pure SnS_2_ electrode. The cation diffusion coefficient can be calculated by following Equation (1):

(1) $$D_{Na^{+}}=\frac{R^{2}T^{2}}{2A^{2}n^{4}F^{4}C^{2}\sigma_{w}{}^{2}}$$

where *R* is the gas constant, *T* is the temperature, *F* is the Faraday constant, *n* is the electron transfer number, *A* is the apparent electrode surface area, and *C* is the maximum sodium ion concentration. The obtained sodium diffusion coefficient of SnS_2_/3DG (1.04 × 10^−14^ cm^2^/s) is much higher than that of the SnS_2_ electrode (1.01 × 10^−15^ cm^2^/s). The good conductivity and fast ion diffusion coefficient improve the electrochemical performance of the SnS_2_/3DG composite.

## 4. Conclusions

In summary, a highly conductive graphene aerogel anchored with SnS_2_ composite was successfully prepared by combining hydrothermal and high-temperature reduction methods. The reversible capacity of the SnS_2_/3DG composite can reach 430 mAh/g at a current density of 100 mA/g after 50 cycles. The resistance of the SnS_2_/3DG composite can be effectively reduced by introducing the conductive graphene network with a high reduction degree, which is beneficial to improving its rate performance. The SnS_2_/3DG composite delivers an outstanding rate capability with the current density increasing from 100 mA/g to 2 A/g. This three-dimensional porous SnS_2_/3DG anode shows significant potential for the next-generation of SIBs.

## Figures and Tables

**Table 1:** The comparison of metal chalcogenides’ electrodes.

Systems	Capacity	Cycle Stability	R_total_ = Re + Rsf + Rct (Ω)	Reference
3D SnS_2_/rGO	0.1 A/g–754 mAh/g 2.0 A/g–401 mAh/g	75.4%	150	Ref. [36]
Exfoliated SnS_2_/Graphene	0.2 A/g–650 mAh/g 4.0 A/g–326 mAh/g	66.6%	100	Ref. [37]
Flower-like SnS_2_/rGO	0.05 A/g–521 mAh/g 0.4 A/g–200 mAh/g	83.3%	400	Ref. [26]
Free-standing SnS_2_/carbon nanofibers	0.2 A/g–570 mAh/g 5.0 A/g–247 mAh/g	81%	/	Ref. [12]
2D SnS_2_/CNTs hybrid	0.05 A/g–476 mAh/g 3.2 A/g–265 mAh/g	84.0%	100	Ref. [38]
NCNF/MoSe_2_	0.5 A/g–386 mAh/g 10.0 A/g–285 mAh/g	91%	300	Ref. [39]
MoS_2_/3DG	0.1 A/g–455 mAh/g 2.0 A/g–310 mAh/g	80.0%	75	Ref. [40]
SnS_2_/3DG	0.1 A/g–498 mAh/g 2.0 A/g–254 mAh/g	67.0%	230	This work
